# A Case Report on Parosteal Lipoma Stretching the Posterior Interosseous Nerve Without Causing Palsy

**DOI:** 10.7759/cureus.14776

**Published:** 2021-04-30

**Authors:** Rishab C, Dilipkumar Naidu, Santhosh Ravi, Dhinakaran S

**Affiliations:** 1 Orthopaedic Surgery, SRM Institute of Science and Technology, Chengalpet, IND

**Keywords:** parosteal lipoma, posterior interosseous nerve

## Abstract

Lipomas are benign soft tissue tumors that can be either superficial or deep. Superficial lipomas are mostly asymptomatic whereas deep-seated lipomas can occasionally cause symptoms if they grow adjacent to neurovascular structures. In this report, we present a case of parosteal lipoma of the right proximal forearm in a 47-year-old male, which was initially diagnosed as intramuscular lipoma stretching posterior interosseous nerve (PIN), with no neurological complaints during both preoperative and postoperative periods.

## Introduction

Lipomas are the most common form of benign soft tissue tumors. They mostly occur in the superficial form in the subcutaneous tissues, which are called superficial lipomas. If they occur beneath the fascia, they are called deep lipomas. Deep lipomas can be intramuscular, intermuscular, and rarely, parosteal lipomas. All superficial lipomas are usually asymptomatic whereas deep-seated lipomas can occasionally cause symptoms if they are located near a nerve or blood vessel. In a study by Sanaa et al., lipoma was assumed to cause monomelic hypertrophy in a young female patient [1].

Even though it has been widely researched and studied, the definitive cause/etiology of lipomas has not been properly defined or established. There are various theories hypothesized on the etiology of lipomas, such as hormonal influence [1,2], trauma, local inflammation, problems in the differentiation of neurogenic and myogenic factors, local muscle degeneration (as suggested by Mori et al. in 2004); all these conditions/factors can lead to the initiation or growth of mature adipocytes, leading to lipoma.

In this report, we discuss the case of a 47-year-old male who presented with a deep lipoma of the right proximal forearm from the supinator muscle almost fraying the posterior interosseous nerve (PIN) but with no neurological deficit. His postoperative period was also uneventful with full neurological functions of the right wrist and fingers.

## Case presentation

A 47-year-old male with right-hand dominance, a farmer by occupation, presented with complaints of pain and swelling over the right forearm for the past year. The patient had been apparently asymptomatic one year ago. Subsequently, he had developed a swelling in the right forearm on the posterolateral aspect, which had been insidious in onset and gradually progressive in nature. It was associated with pain, aggravated by physical activities, and relieved completely when at rest. He had difficulty performing his routine activities because of the pain.

**Figure 1:**
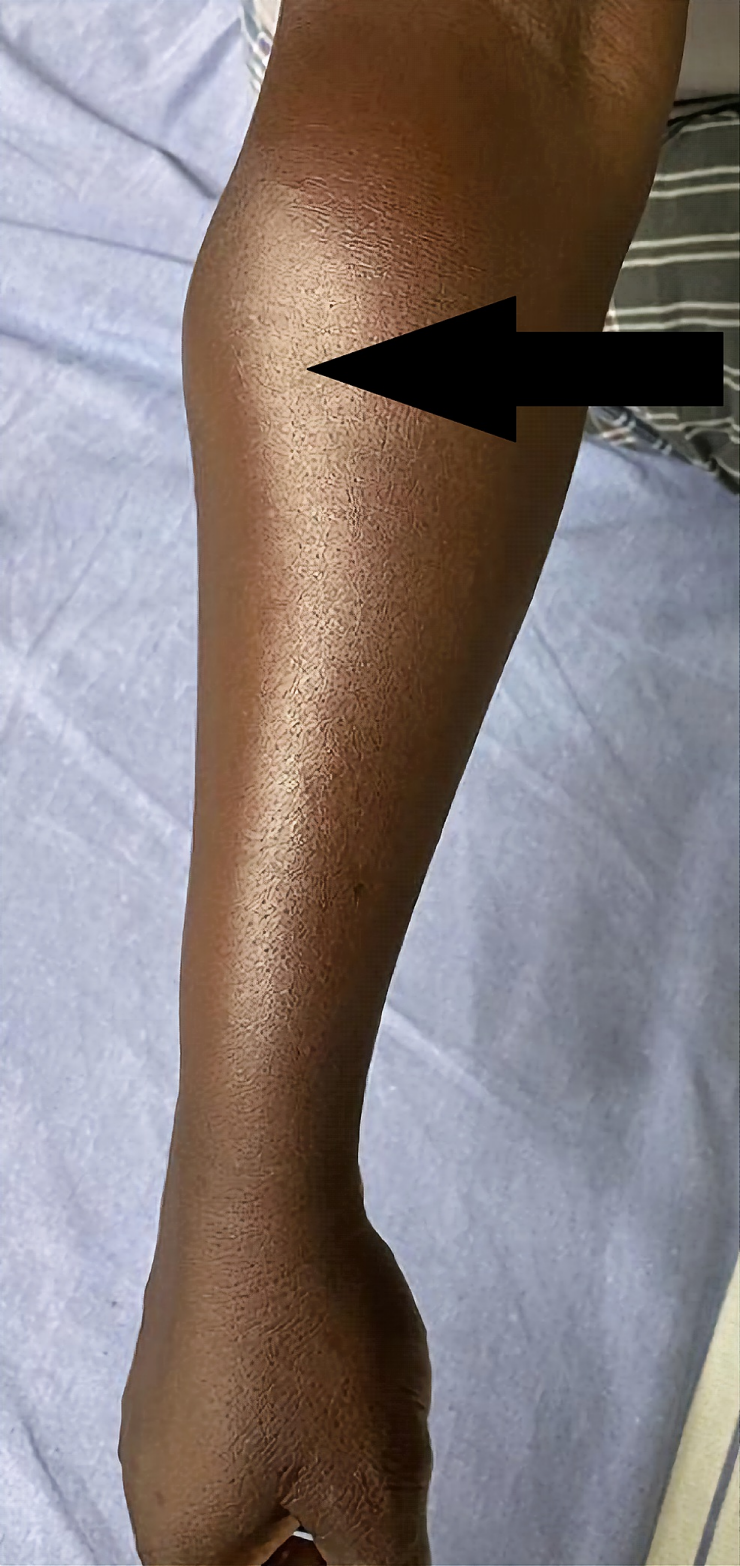
Swelling on the posterolateral aspect of the right proximal forearm (arrow)

**Figure 2:**
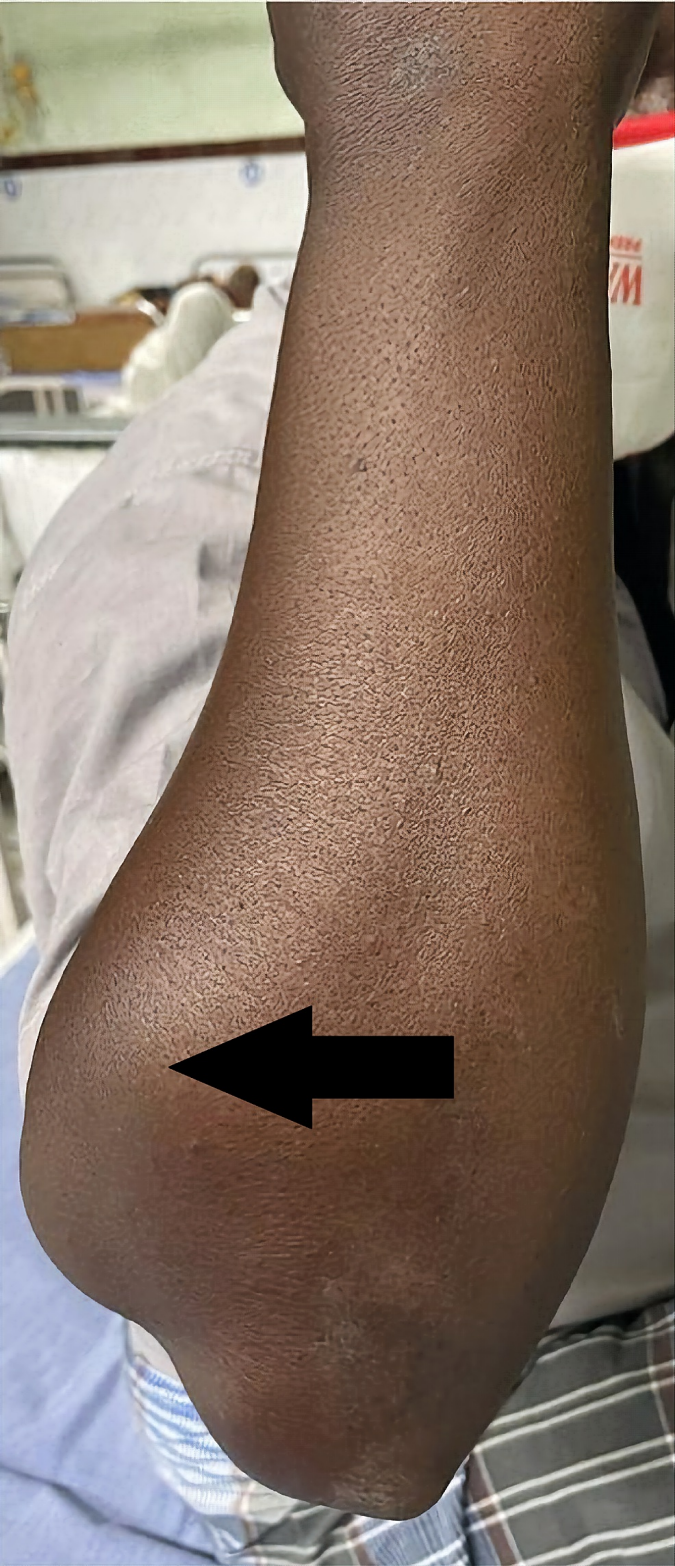
Swelling of the proximal forearm seen from the dorsal aspect (arrow)

An examination of the right forearm revealed swelling of 10 x 5 cm in size over the posterolateral aspect of the proximal forearm just below the elbow flexion crease (Figures 1, 2). The swelling was firm in consistency with smooth and round margins. There were no dilated or engorged veins, scar, or sinus. The swelling was neither warm nor tender. The plane of mobility could not be assessed due to the size and site, but it was not adhered to the skin. On contraction of the forearm muscles, there was no change in the size of the swelling. The elbow movements were normal. Wrist and finger extensions were also normal. The radial pulse was palpated and appeared the same compared to the left side.

**Figure 3:**
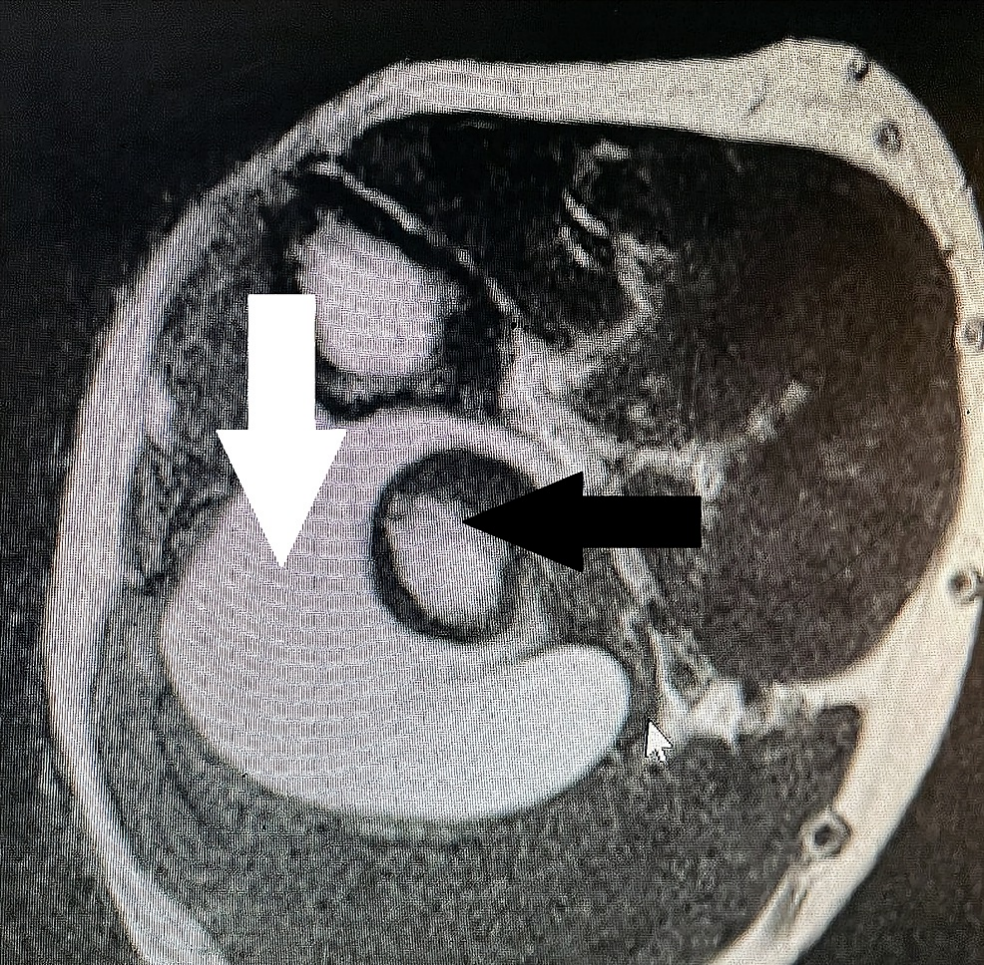
MRI (axial section) of the right forearm showing the circumferential tumor (white arrow) around proximal radius (black arrow) MRI: magnetic resonance imaging

**Figure 4:**
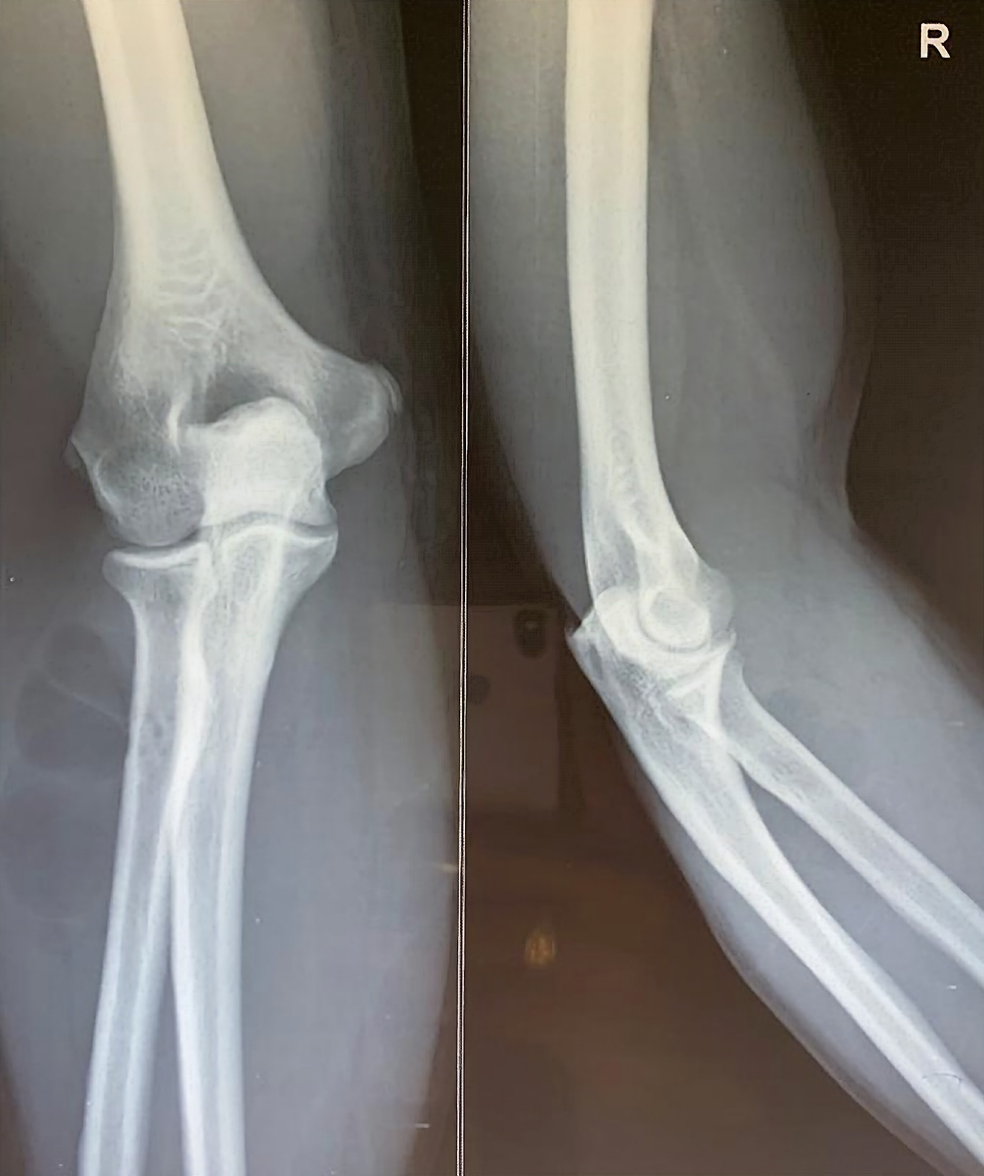
Plain X-ray of the right elbow with the forearm

**Figure 5:**
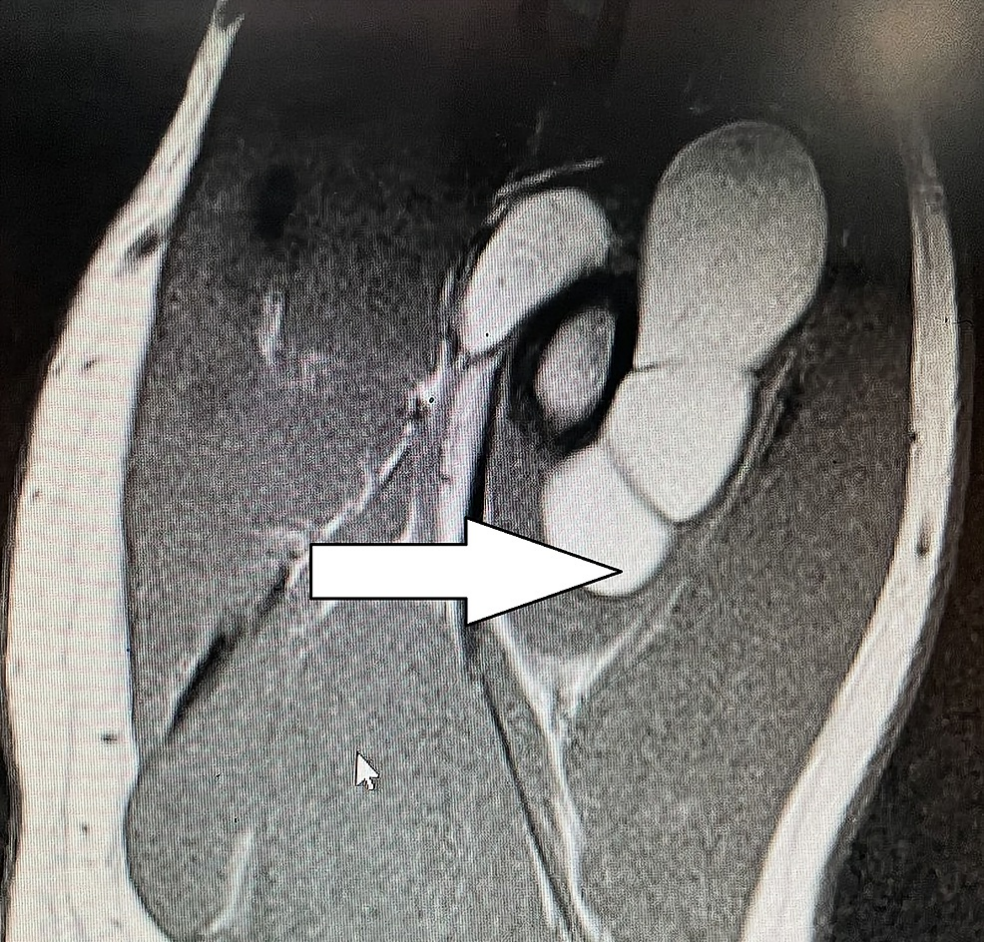
MRI showing the extent of the tumor (arrow) MRI: magnetic resonance imaging

Plain X-ray of the right elbow and forearm showed tiny holes in the proximal aspect of the radius with scalloping of the lateral cortex in the anteroposterior view with soft tissue shadows with septations adjacent to the proximal radius (Figure 3). In view of the soft tissue tumor, an MRI of the forearm was performed (Figures 4, 5). The report showed lobulated intramuscular lesion, which was hypointense on T1-weighted sequence within the supinator muscle with extension into the intramuscular plane of the flexor aspect, abutting the neurovascular bundle on the flexor aspect with no signal changes of the PIN, and with no signal changes in the bone.

**Figure 6:**
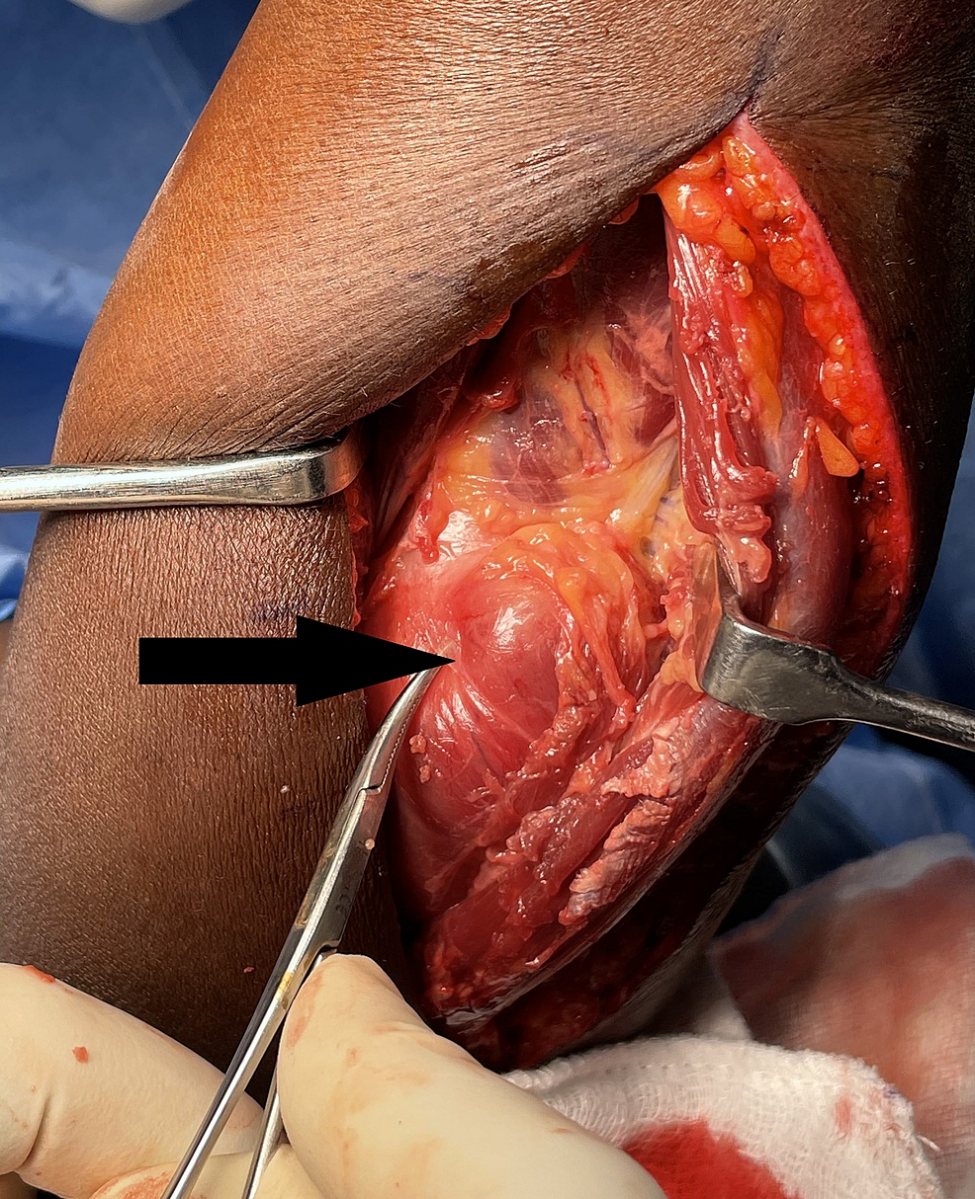
Posterolateral (Thompson) approach showing supinator (arrow) with a mass

**Figure 7:**
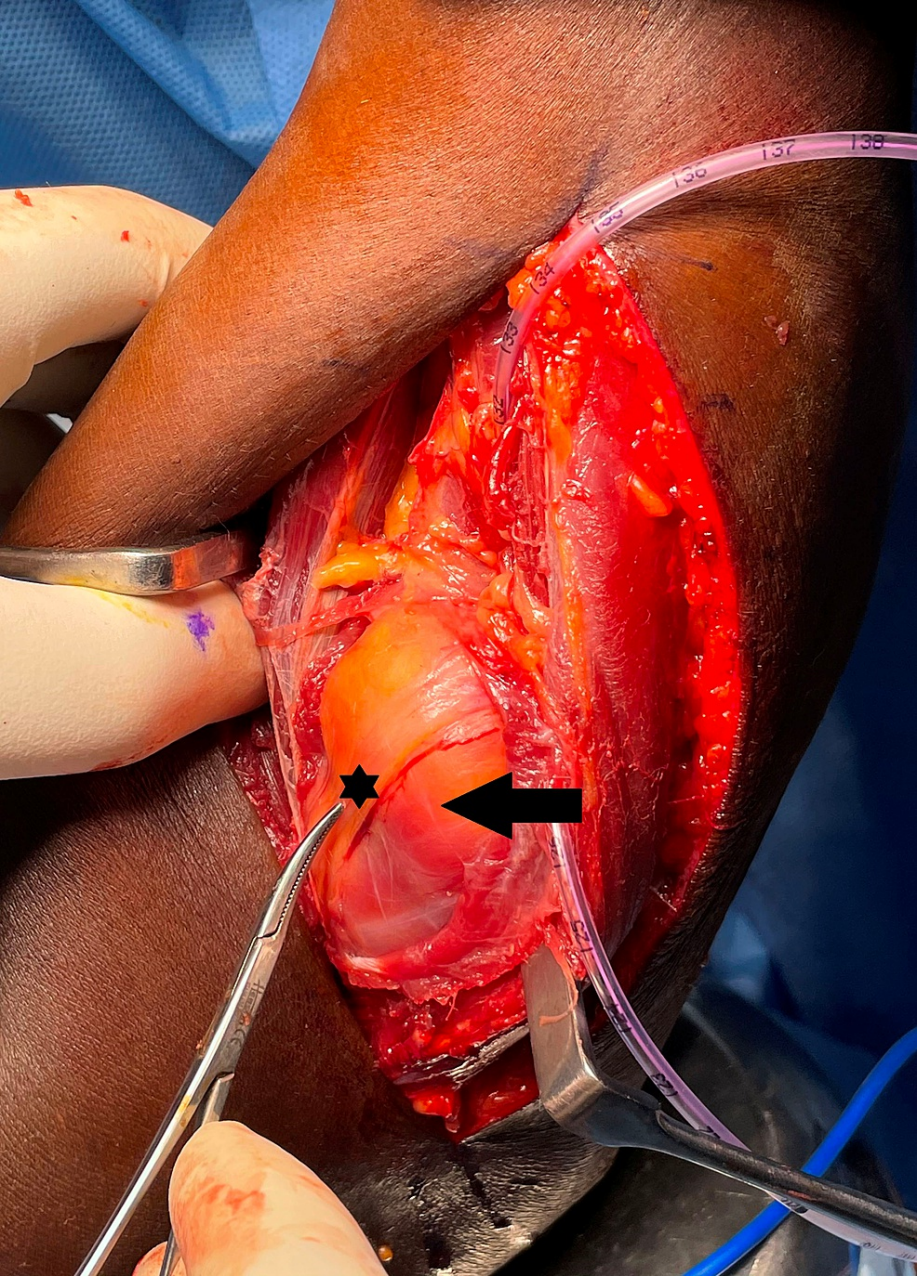
Thompson approach showing the lipoma (arrow) and PIN (star) being stretched by the mass PIN: posterior interosseous nerve

**Figure 8:**
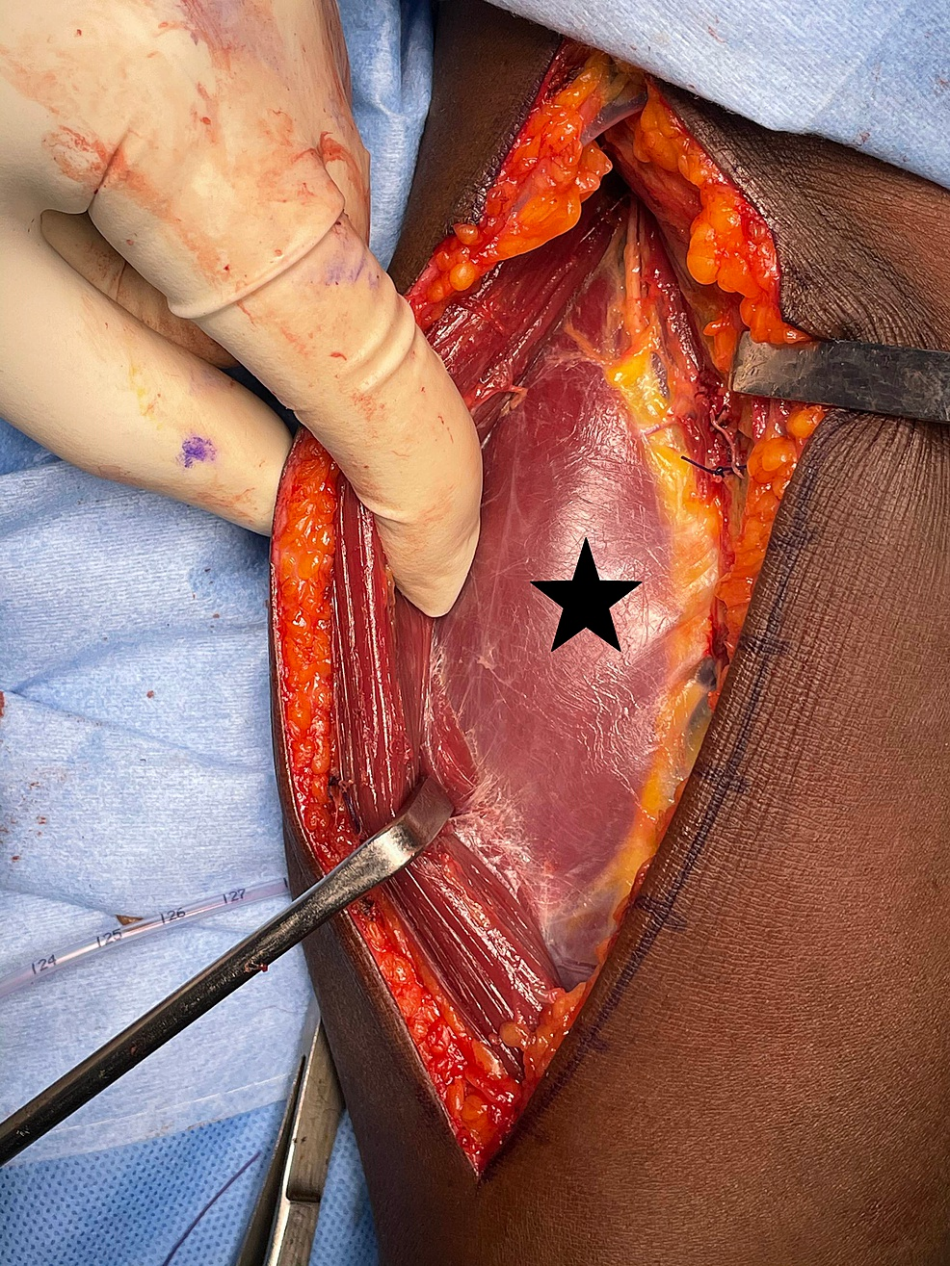
Volar (Henry) approach showing supinator muscle (star)

**Figure 9:**
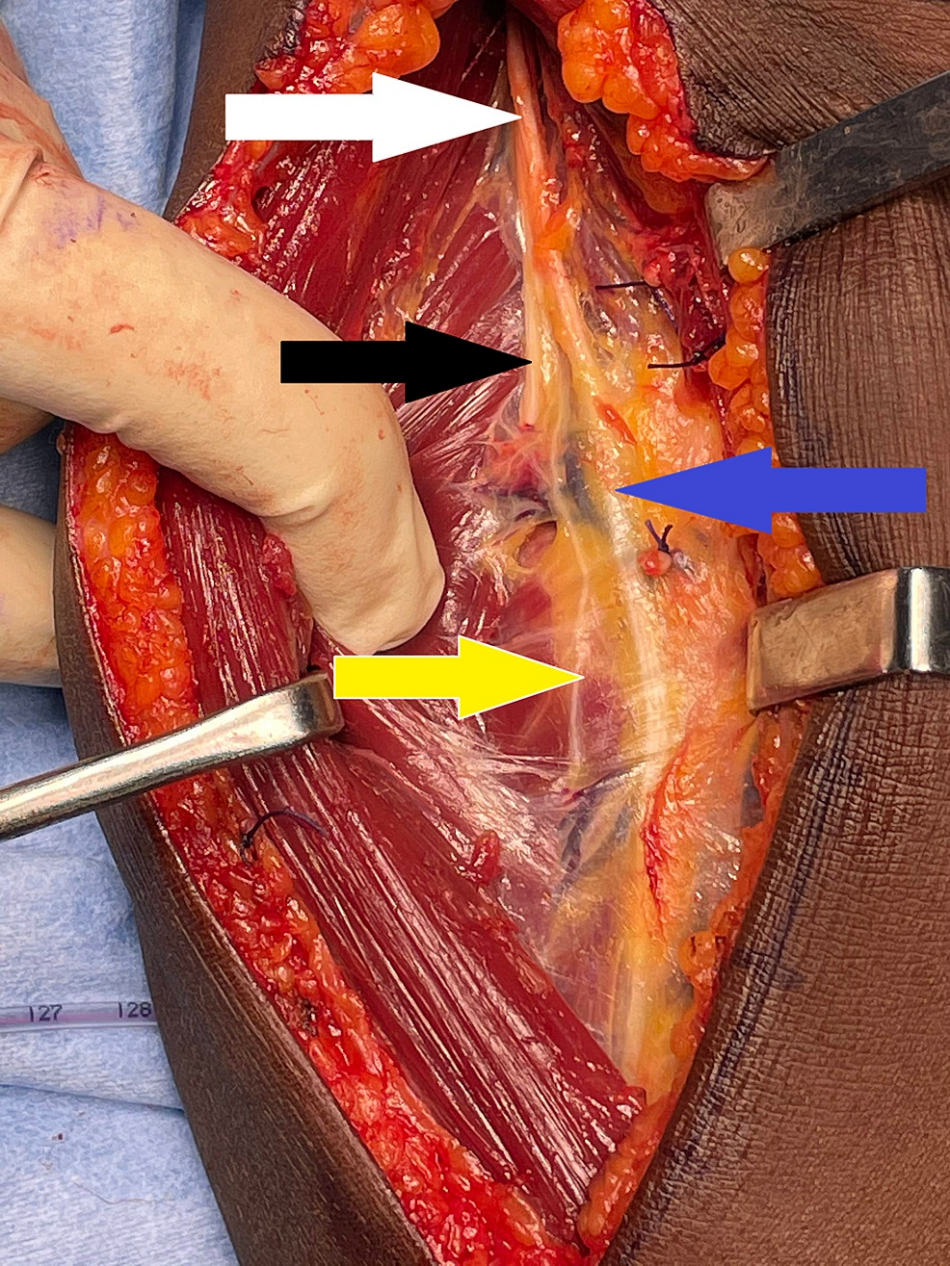
Approach extended to track radial nerve (white arrow) and its branches: superficial radial nerve (blue arrow), nerve to extensor carpi radialis brevis (yellow arrow), and PIN (black arrow) PIN: posterior interosseous nerve

**Figure 10:**
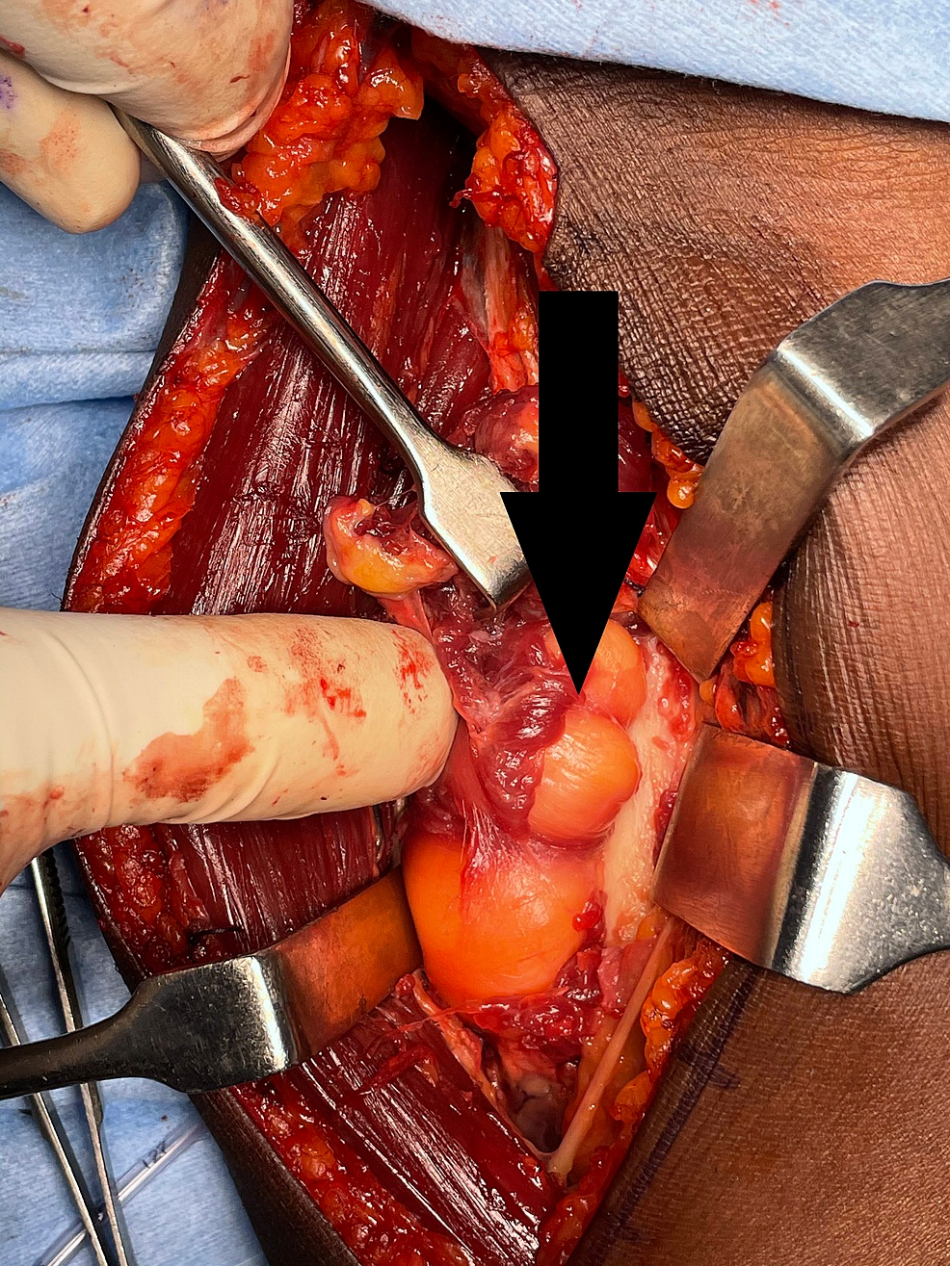
Lipoma (arrow) seen through the volar approach

**Figure 11:**
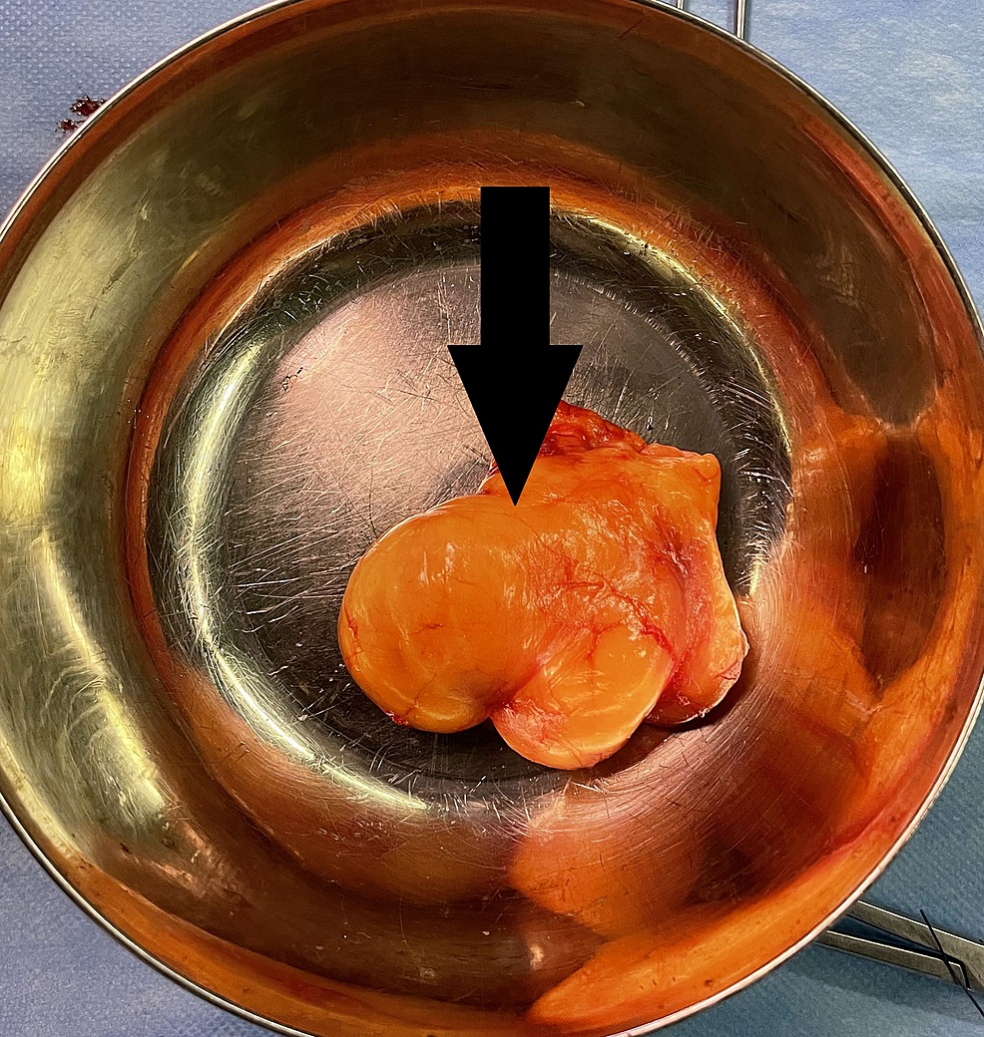
Excised lipoma (arrow)

**Figure 12:**
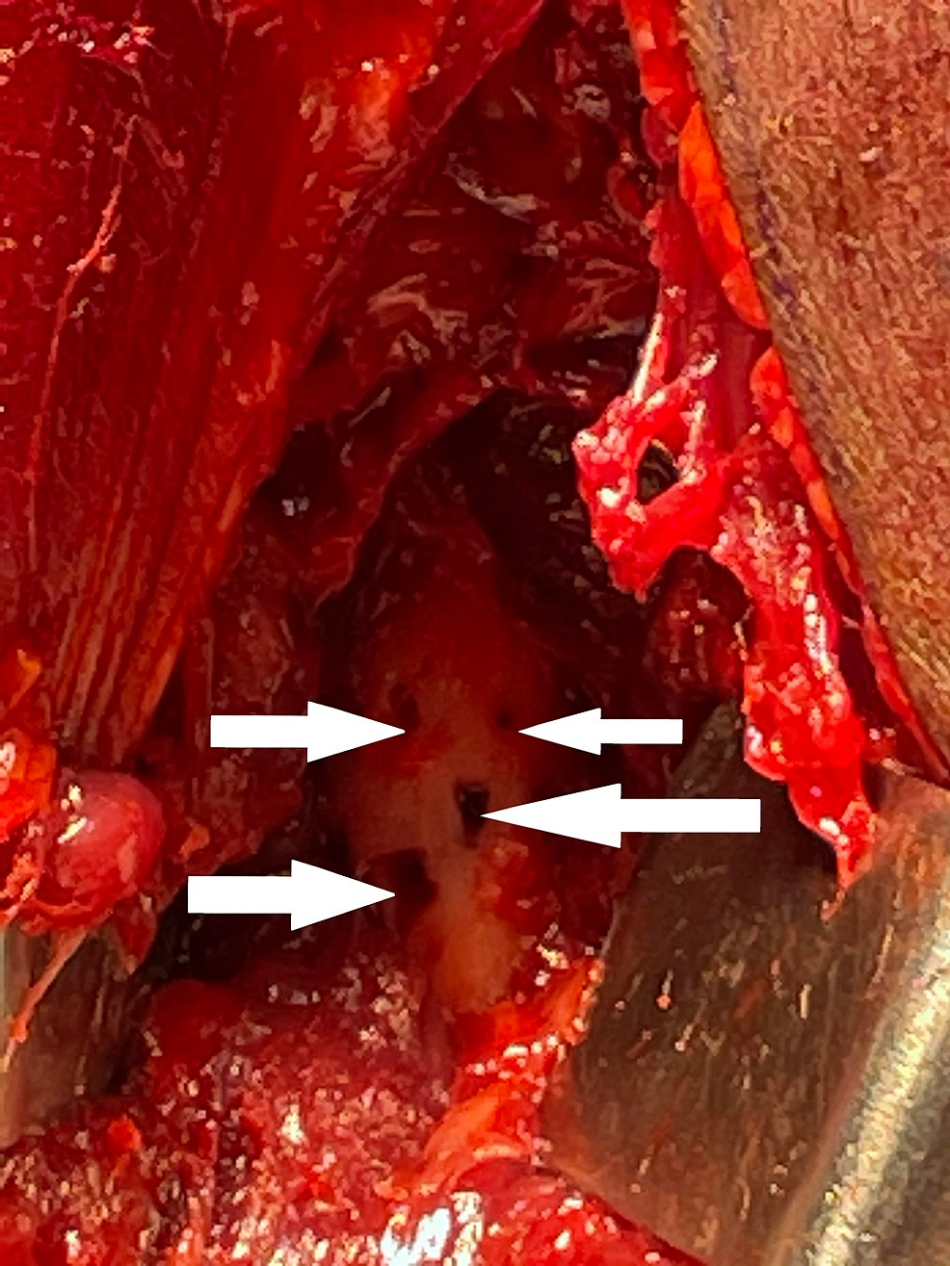
Holes (arrows) seen in the proximal radius where the lipoma was adherent

**Figure 13:**
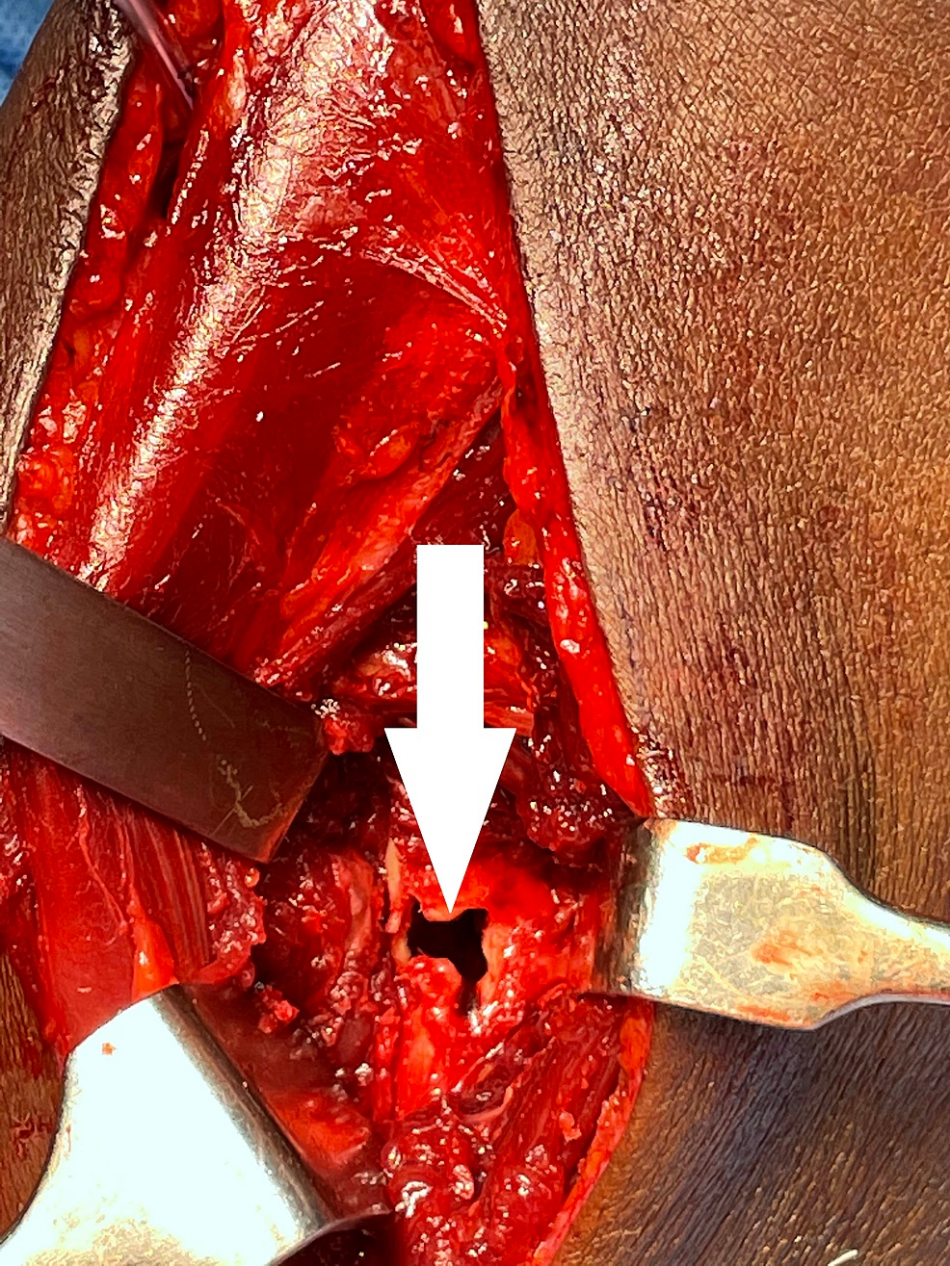
Debrided and curetted holes (arrow)

After counseling about PIN palsy, the patient was taken for surgical exploration and excision of the lipoma. The Thompson approach was planned to handle the tumor from the posterolateral aspect. With the patient in the supine position, under strict sterile precautions, through a posterolateral (Thompson) approach, and with the plane of dissection made between extensor carpi radialis brevis and extensor digitorum communis, the mass was identified within the supinator muscle (Figure 6). After careful dissection of the supinator muscle, the PIN was identified, which appeared stretched and frayed (Figure 7). As the mass was circumferential, as noted on the MRI, and since the PIN wound around the neck of radius, we had to use the Henry approach. The dissection was made between brachioradialis (BR) and pronator teres to reach the supinator muscle (Figure 8). The supinator muscle was stretched substantially with the mass underneath. The incision and soft tissue dissection extended even above the elbow crease, and a plane was made between BR and brachialis to trace the radial nerve and track down its branches (Figure 9). The mass was identified within the supinator (Figure 10), and after careful dissection on both anterior and posterior aspects of the proximal radius, the mass was excised in toto, and the tumor was found to measure about 4 x 4 x 6 cm (Figure 11). Also, the proximal radius near the neck had four holes (Figure 12), where the mass was adherent. The holes were curetted with the stripping of the periosteum (as per Salama et al., 2021) (Figure 13). The excised mass was sent for histopathological examination (HPE). Again, the PIN was inspected, and it appeared to be intact but stretched and attenuated as shown in Figure 7. The wound was washed and closed in layers.

**Figure 14:**
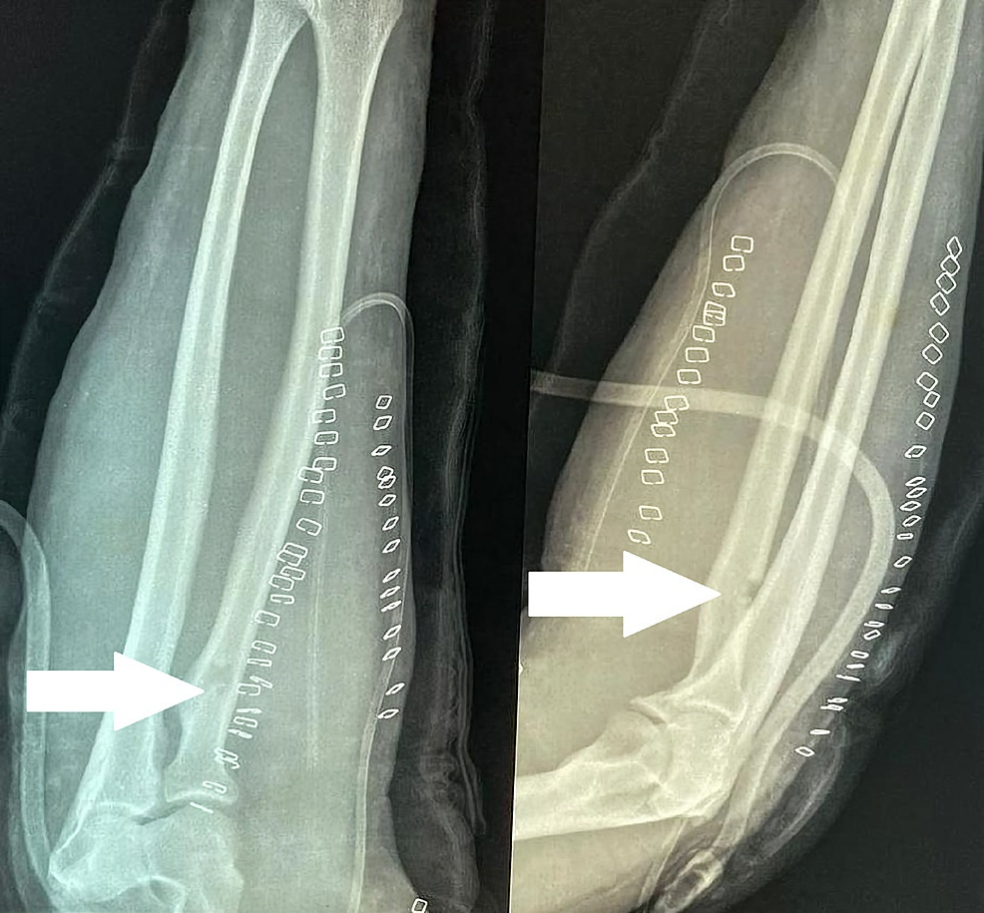
Immediate postoperative X-ray of the right forearm showing cortical breach (arrows) in proximal radius where the curettage was done

Postoperatively, the patient was found to have full neurological functions. Plain X-ray in the immediate postoperative period showed cortical breach (Figure 14) where the holes were curetted; otherwise, it was normal. The limb was not immobilized as the breach in the cortex post curettage was less than 30% of the circumference of the proximal radius. The patient's wrist and finger extension on the right side was 5/5 as per the Medical Research Council grading for muscle power. Sutures were removed at the end of the second week, and the patient returned to work after 12 weeks with frequent follow-ups. The HPE report showed mature adipocytes with some osseous matrix found scattered eccentrically, which was consistent with parosteal lipoma, and it grossly showed yellowish capsulated lesion.

## Discussion

There have been numerous reports on lipoma causing PIN palsy in the literature. But this report is on a fortunate patient who had no clinical symptoms or signs of PIN palsy though the lipoma was seen stretching the PIN. This patient had not lost many workdays due to the condition. Hence, we emphasize on the importance of earlier investigation and treatment for this benign tumor. Lipomas are benign soft tissue tumors that originate from mature adipocytes. Their malignant counterpart is actually thought to be a separate entity [3]. Intramuscular lipoma was first reported in 1856 in the trapezius muscle by Paget [1,4]. Lipoma causing PIN palsy was first reported by Richmond in 1953 [5,6]. Other non-traumatic causes that can lead to PIN palsy are ganglionic cyst in the proximal forearm (first reported by Boven and Stone in 1966), synovial cysts, chondroma, fibromas, arterio-venous malformations, neuralgic amyotrophy [7], supinator entrapment or focal nerve constrictions [8], etc. In rare cases, blunt trauma attributed to inflammation of muscle can also lead to lipoma [9].

The radial nerve arises from the posterior cord from C5 to T1 of the brachial plexus. It divides into superficial and deep branches. The superficial radial nerve is sensory to the dorsal and lateral aspects of the hand. The deep branch, also known as the PIN, is embedded between the two heads of the supinator muscle. It is motor to the wrist and finger extensors. This branch winds around the proximal part of the radius, and this unique anatomical relation makes the PIN prone to palsy even due to trivial stretching in that region by any of the above-mentioned lesions or even iatrogenic factors while addressing proximal radius fractures.

Most soft tissue tumors, especially lipomas, can be diagnosed clinically, but an MRI is required to confirm the diagnosis, which shows its complete extension. The nerve integrity can be seen well with signal changes on MRI using diffusion tensor imaging [10]. Diffusion-weighted MR neurography (DW-MRN) [11] has also been used, which uses a unique software to subtract other tissues and visualizes the nerve. Ultrasonography can also be used to visualize lipoma causing PIN palsy [6]. In case of nerve deficits, nerve conduction studies can reveal the region of nerve injury. Surgical excision is recommended for PIN palsy recovery in cases of lipoma near to proximal radius [12]. The recovery in cases of PIN palsy takes around six weeks to three months [6,13,14], and the longest recovery time reported is 24 months [15].

Superficial lipoma situated in the subcutaneous tissues is asymptomatic, and patients usually seek medical intervention only due to cosmetic reasons, whereas deep lipomas can cause symptoms, and patients seek intervention for pain or neurovascular compromise. Deep lipomas are mostly infiltrative lipomas that involve the muscles and have incomplete capsules [16], and recurrence has been reported in such cases [17]. Some authors reserve the term infiltrating lipoma if it involves the adjacent structures [3]. Indications for surgical excision of lipomas can be growing tumors (size of >5 cm), close proximity to the neurovascular bundle, subfascial locations, pain, and cosmetic reasons [1]. In a literature review by Serpell et al., the median size of deep lipomas excised was 11 cm [18]. Surgery for parosteal lipomas has shown excellent results [12].

In cases of circumferential soft tissue tumors in the bone or areas where neurovascular bundles are in close proximity to the tumors, 3D reconstruction of MRI images using Mimics computer-aided design (CAD) (Materialise NV, Leuven, Belgium) has been used before surgical excision [19]. Normally, 3D images are available for CT, but here they used MRI images as CT does not visualize soft tissue tumors.

Though surgical exploration and excision have shown good results, recurrences have been reported, which range from 8-62.5% of cases [20]. Most of the recurrences are attributed to inadequately excised margins, and recurrence can be avoided by using combined margin and wide excision techniques [17].

## Conclusions

The unique anatomy of PIN at the proximal forearm makes it vulnerable to palsy due to any lesions in that region, most commonly lipoma as in our case. Surgical exploration and decompression can prevent PIN from getting injured if diagnosed early. In this case, we opted for a combined dorsal and volar approach in order to excise the mass in toto along with the periosteum surrounding it and also to track the PIN above and below the mass. Even if the patient develops PIN palsy, decompression and excision can provide complete recovery as discussed above.
